# Left Ventricular Lead Placement for Biventricular Pacing in a Left Lateral Vein in a Patient with Congenital Atresia of the Coronary Sinus Ostium

**DOI:** 10.19102/icrm.2023.14021

**Published:** 2023-02-15

**Authors:** Amato Santoro, Maria Barilli, Nicolò Sisti, Alex di Florio, Claudia Baiocchi, Luigi Muzzi, Eugenio Neri

**Affiliations:** ^1^Division of Cardiology, Azienda Ospedaliera Universitaria Senese, Siena, Italy; ^2^Division of Cardiac Surgery and Aortic Disease, Department of Cardio-thoracic Surgery, Azienda Ospedaliera Universitaria Senese, Senese, Italy

**Keywords:** Challenge biventricular pacing implantation, coronary sinus atresia, left persistent cava vein

## Abstract

Coronary sinus ostial atresia (CSOA) is a rare kind of congenital heart anomaly. This creates a new drainage pathway for the cardiac venous flow, with the most common being a persistent left superior vena cava (PLSVC). During the implantation of a cardiac resynchronization therapy defibrillator, we discovered a case of CSOA in a patient who underwent aortic valve and ascending aorta replacement. CSOA led to the research and subsequent identification of a PLSVC, which drained in the CS. The left ventricular pacing lead was appropriately placed in a left lateral vein. This case report highlights the technical aspects and procedural difficulties that characterize this specific anatomical variant.

## Introduction

Coronary sinus ostial atresia (CSOA) is a kind of congenital heart anomaly with an incidence rate of 0.1%–0.25%.^1^ In affected patients, the CS ostium is occluded and does not drain into the right atrium, forcing the venous flow to find a different pathway for cardiac drainage. This anatomic variant is commonly associated with a persistent left superior vena cava (PLSVC), which becomes the new passage for the blood flow to reach the right atrium. In our case report, we describe how CSOA was approached after an accidental finding during the implantation of a cardiac resynchronization therapy defibrillator (CRT-D) in a patient who underwent aortic valve and ascending aorta replacement.

## Case presentation

**Figure 1:**
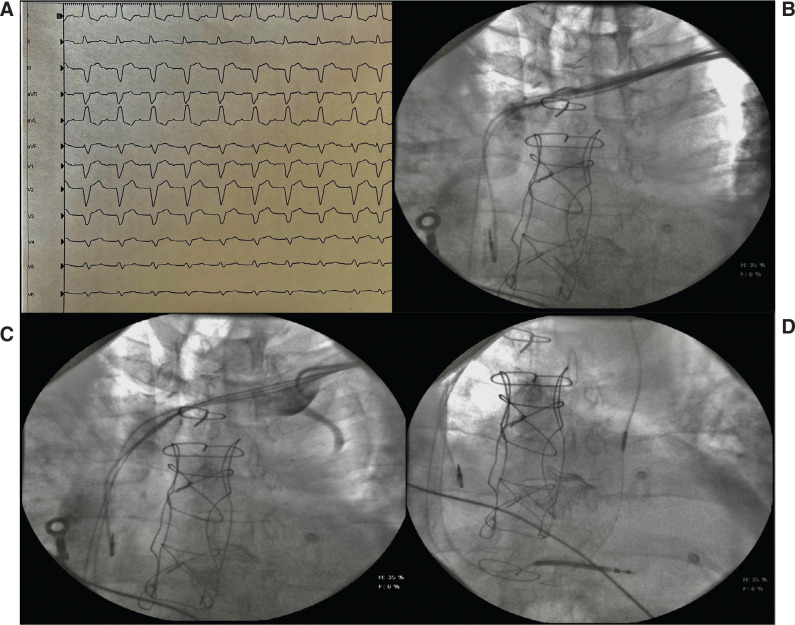
**A**: Basal electrocardiogram: I degree, type II atrioventricular block and left bundle branch block. **B**: Injection of contrast in the superior vena cava from delivery. **C**: A small notch in the left subclavian venous flow revealed the existence of an inflow during contrast injection in the persistent left superior vena cava (PLSVC). **D**: A guidewire was inserted in the PLSVC.

A 66-year-old man suffering from sarcoidosis came to our attention after experiencing an episode of syncope under exertion. His medical history included a previous hospitalization for chest pain, during which he was assessed for a suspicion of aortic dissection. Computed tomography angiography was performed, revealing an aortic root dilation of 40 mm and an ascending aortic aneurysm with an anteroposterior diameter of 50 mm. Moreover, lesions indicative of pulmonary sarcoidosis were found. The electrocardiogram (ECG) recorded at hospital admission showed normal sinus rhythm, a first-degree atrioventricular block (PR, 230 ms), and left bundle branch block (LBBB) with a wide QRS interval (170 ms) **(Figure 1A)**. Transthoracic echocardiography was also performed, showing left ventricle (LV) dilatation with severe systolic dysfunction, inferior LV wall akinesia and dyskinesia due to LBBB (ejection fraction [EF], 33%), and increased transaortic gradient (Gmed, 38 mmHg) compatible with severe low-flow, low-gradient aortic stenosis accompanied by moderate aortic insufficiency. During hospitalization, the patient was subjected to further comprehensive tests, including cardiac magnetic resonance (CMR) imaging, which confirmed cardiac involvement of the systemic granulomatous disease that was previously suspected. Preoperative coronary angiography showed coronary atherosclerosis in the absence of significant lesions.

Given the finding of severe valvulopathy together with an ascending aortic aneurysm, the patient was submitted to surgery with efficacy. A bioconduit connected with the aortic valve replacement reconstituted the ascending aorta. Postoperative ECG monitoring showed no pauses, but various episodes of type 1 Luciani–Wenckebach and type 2 second-degree atrioventricular block (AVB) were documented. In consideration of the severe LV dysfunction, confirmed by a heart CMR exam that revealed an EF of 33%, with a QRS duration of 170 ms in the context of LBBB, the patient underwent CRT-D implantation for the primary prevention of sudden cardiac death on the 10th postoperative day.

### Description of the procedure

**Figure 2:**
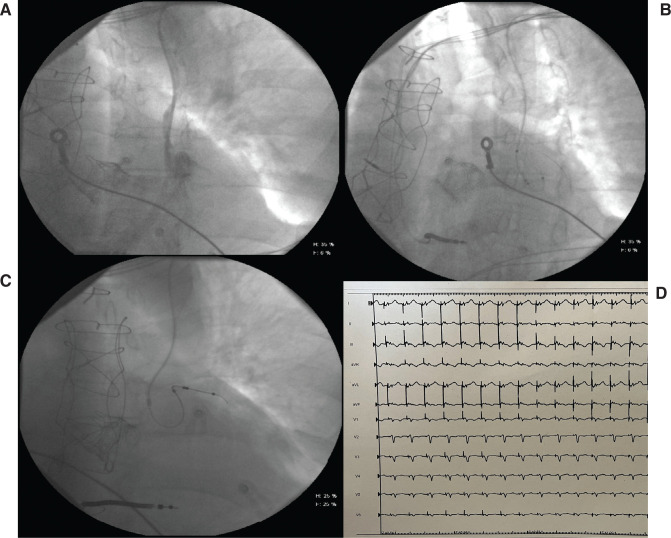
**A**: Contrast injection of the persistent left superior vena cava (PLSVC) demonstrating connection to oblique vein of the left atrium and then the coronary sinus. **B**: The subselect catheter is retracted in the PLSVC and a quadripolar catheter was prolapsed in the ostium of the lateral target vessel, with a guidewire advanced distally to the lateral vein. **C**: Final position of the quadripolar coronary sinus lead in the lateral vein. **D**: Final electrocardiogram.

An incision was made at the level of the left deltopectoral groove, 2 cm below and parallel to the left clavicle, after local anesthesia (lidocaine 2%). Once the left cephalic vein was surgically isolated, a right ventricular cardioverter-defibrillator lead and an atrial pacing lead were placed on the lower apical interventricular septum and the atrial septum, respectively. Extrathoracic subclavian vein puncture was then performed, using the Seldinger technique, and a 9-French valved introducer was positioned. The right atrium was probed using a quadripolar torque catheter (Medtronic Inc., Minneapolis, MN, USA) to cannulate the CS ostium. After numerous attempts, a mild injection of contrast medium was performed to visualize the CS inlet, but no communication was revealed. After this ineffective attempt, the quadripolar electrophysiologic catheter was connected to the polygraph and the entire tricuspid valve was mapped, guided by endocavitary signals, but no connection between the CS and the right atrium was cannulated. The superior and inferior vena cavae were then analyzed with a delivery system and quadripolar torque lead to find possible anatomical variations. The quadripolar lead was removed and contrast medium was administered from the superior vena cava to the distal left subclavian vein during delivery system extraction in order to visualize the PLSVC, as shown in **Figure 1B**. A small notch in the left subclavian venous flow revealed the existence of an inflow. Once the delivery was removed, the ostium was cannulated using a subselector (Attain Select 2; Medtronic) with an angle of 130° to cannulate the PLSVC. The subselector was successfully introduced though the PLSVC, and a hydrophilic guidewire (180; Terumo Interventional Systems, Somerset, NJ, USA) was used to select the vessel **(Figure 1C)**. Using the subselector with the over-wire technique, it was possible to visualize the CS, which appeared normal in size without a connection in the right atrium **(Figure 1D)**. Contrast medium injection was performed while retracting the catheter, and a left lateral vein originating from the CS was highlighted **(Figure 2A)**. A guidewire (Thunder; Medtronic) was used for cannulation of the lateral vein ostium, and a subselector sheath was introduced into the vein inlet. Lateral vein cannulation was first performed with a Medtronic Cougar XT guidewire, and a quadripolar lead (Attain Performa 4598; Medtronic) was advanced over the wire with unsuccessful cannulation **(Figure 2B)**.

A second attempt was made with the Choice Floppy Guidewire (0.014 in, 182 cm) from Boston Scientific Corp. (Marlborough, MA, USA), and the first curve of the lateral venous ostium was engaged after a counterclockwise rotation of the subselector sheath over the wire, as visualized with selective venography. Once the vein was cannulated, the subselector was advanced beyond the acute angle and retracted proximally toward the inlet of the lateral vein. The guidewire was tensioned distally, and after many attempts to push and pull the lead over the wire while retracting the guide inside the LV lead, it was successfully inserted along the left lateral vein **(Figure 2C)**. With a dedicated cutter, the subselector was removed without displacement of the electrodes. A guidewire was then reintroduced distally to the quadripolar catheter with effective reduction of the lead loop. Tip-to-tip conduction delays from the right ventricular lead to the distal poles of the left lateral vein lead were then analyzed. A spontaneous right ventricle-to-LV electrogram delay of 130 ms, right ventricle paced-LV sensed delay of 130 ms, and LV paced-right ventricle sensed delay of 120 ms were reported (LV lead threshold, 1 V × 0.5 ms). A QRS duration of 90 ms was obtained during biventricular pacing stimulation according to the ECG **(Figure 2D)**. All leads were finally connected to a Medtronic CRT-D Amplia device battery and fixed to the muscular plane. After 4 months, the EF was increased to 45%.

## Discussion

The CS is an important anatomical structure as it represents the gateway to many electrophysiological and cardiac surgery procedures. It is important to identify the presence of possible anatomical abnormalities in order to properly plan every scheduled step. CSOA is a rare congenital anomaly often associated with a PLSVC.^1^ This anatomic variant leads to major limitations when the placement of a LV lead is needed during a CRT procedure.^2^ This case report describes a possible technique for placement of the LV lead through subclavian access when there is a suspicion of CSOA. It represents an alternative to epicardial positioning of the left lead after thoracotomy. The insertion of a LV lead through a persistent left vena cava is complex and requires the use of different materials compared to a traditional approach. When CSOA is suspected, it is fundamental to study the venous system both with venography and, if possible, with pre-procedural imaging methods such as computed tomography angiography to prevent procedural failure. This procedure was recently described by Bajwa et al. in 2021.^3^ Zou et al. also reported a series of patients with the combination of CSOA and PLSVC identified at the time of LV lead implantation.^4^ Unlike in their cases, we found a sharper angle to enter the lateral vein that showed a thin gauge in our case. Moreover, to cannulate the lateral vein, we used the Attain Performa 4598 catheter, which has a spiral-like appearance, advancing it through a subselector to ensure lead support over the guidewire. An acute angle was found to enter the lateral vein and progressed with a double angle after the venous ostium, which only allowed for positioning of the guidewire with rejection of the catheter. We modified the procedure for the cannulation by retracting the subselector right before the venous ostium and gradually advancing the lead with a push-and-pull technique. Our case includes the performance of a very complex technique of CS lead placement in a PLSVC in the setting of CSOA.
